# An Integrating Approach for Genome-Wide Screening of MicroRNA Polymorphisms Mediated Drug Response Alterations

**DOI:** 10.1155/2017/1674827

**Published:** 2017-04-05

**Authors:** Xianyue Wang, Hong Jiang, Wei Wu, Rongxin Zhang, Lingxiang Wu, Huan Chen, Pengping Li, Yumin Nie, Jiaofang Shao, Yan Li, Xue Lin, Sali Lv, Qh Wang, Jie Hu

**Affiliations:** ^1^Department of Bioinformatics, Nanjing Medical University, Nanjing 210029, China; ^2^Key Laboratory of Human Functional Genomics of Jiangsu Province, Nanjing Medical University, Nanjing, China; ^3^Collaborative Innovation Center for Cardiovascular Disease, Nanjing Medical University, Nanjing, China

## Abstract

MicroRNAs (miRNAs) are a class of evolutionarily conserved small noncoding RNAs, ~22 nt in length, and found in diverse organisms and play important roles in the regulation of mRNA translation and degradation. It was shown that miRNAs were involved in many key biological processes through regulating the expression of targets. Genetic polymorphisms in miRNA target sites may alter miRNA regulation and therefore result in the alterations of the drug targets. Recent studies have demonstrated that SNPs in miRNA target sites can affect drug efficiency. However, there are still a large number of specific genetic variants related to drug efficiency that are yet to be discovered. We integrated large scale of genetic variations, drug targets, gene interaction networks, biological pathways, and seeds region of miRNA to identify miRNA polymorphisms affecting drug response. In addition, harnessing the abundant high quality biological network/pathways, we evaluated the cascade distribution of tarSNP impacts. We showed that the predictions can uncover most of the known experimentally supported cases as well as provide informative candidates complementary to existing methods/tools. Although there are several existing databases predicting the gain or loss of targeting function of miRNA mediated by SNPs, such as PolymiRTS, miRNASNP, MicroSNiPer, and MirSNP, none of them evaluated the influences of tarSNPs on drug response alterations. We developed a user-friendly online database of this approach named Mir2Drug.

## 1. Introduction

MicroRNAs (miRNAs) are a class of evolutionarily conserved small noncoding RNAs, 19~25 nt in length, and found in diverse organisms [1]. MiRNAs play important roles by binding to the 3′-untranslated region (3′ UTR) of the target mRNA, causing the reduction of its abundance and translational efficiency [2]. It has been shown that miRNAs are involved in many biological processes of human complex diseases through regulating gene expression [3]. Many of the target genes of miRNAs are drug targets, and the genes involved in drug disposition may also be regulated by miRNAs [4]. Therefore, genetic polymorphisms in miRNA target sites may alter the miRNA regulation of these drug-related genes and result in the differential expression of the drug-related protein, which can in turn influence the drug response.

Recently, as the number of drug-related SNPs in miRNA target sites are rapidly increasing, the importance of SNPs positioned in the 3′ UTR regions is becoming evident [5]. Several recent studies have demonstrated that the single nucleotide polymorphisms in miRNA target sites can affect drug efficiency. Mishra et al. showed that a functional SNP presents in 3′ UTR of dihydrofolate reductase, an important drug target, and the SNP interferes with the miR-24 miRNA function and leads to DHFR overexpression and methotrexate resistance [6]. Wynendaele et al. demonstrated that an SNP created an illegitimate miRNA target site within the 3′ UTR of MDM4, which affected ovarian cancer progression and chemo sensitivity [7]. Boni et al. also demonstrated that several SNPs had a significant association with clinical outcome of ovarian cancer patients treated with the 5-FU and CPT-11 combination [8]. Polymorphisms in the miRNA target sites are emerging as powerful tools to elucidate the underlying mechanisms of different responses to treatments in patients. In addition, several other studies indicate that miRNAs can affect drug sensitivity and resistance in cancer chemo therapy [9]. Based on the above-mentioned experimental evidence, we have reason to believe that SNP in miRNA target sites affect drug response may be common, and there are still a large number of these specific genetic variants that have yet to be discovered. However, experimental approaches to identify these SNP that can affect drug response are labor-intensive and time-consuming. To address these challenges, computational and analytical tools can be developed to provide the successful design of biological experiments and interpretation of the results. Characterization of SNP in miRNA target sites in drug response helps predict patients' responses to drug treatments, guides rational drug use, and improves drug safety and efficacy.

Several studies have developed databases or tools that can predict SNPs reside in miRNA target sites. Bao et al. developed a database that collected naturally occurring DNA variations in putative miRNA target sites; this database integrates sequence polymorphism, phenotype, and expression microarray data [10]. Hiard et al. developed a database that compiled DNA sequence polymorphisms that are predicted to perturb miRNA mediated gene regulation. The database also includes the inclusion of copy number variants and eQTL information that affect miRNA precursors as well as genes encoding components of the silencing machinery [11]. Barenboim et al. developed a web-based tool that predicted the impact of an SNP on putative miRNA targets [12]. This application interrogates the 3′-untranslated region and predicts if an SNP within the target site will disrupt/eliminate or enhance/create a miRNA binding site. Hariharan et al. analyzed SNPs in and around predicted miRNA target sites; polymorphisms within 200 nucleotides that could alter miRNA regulation were annotated [13]. However, all existing tools above did not concern the correlation between polymorphisms in miRNA target sites and drug response. More specifically, in addition to variation located in the target site, variation near the target site has been identified as another crucial factor that can influence an individual's response to drugs. For example, an SNP is located 14 bp downstream of the miR-24 target site in DHFR 3′ UTR, does not directly fall within the miRNA target set, and resulted in DHFR overexpression and MTX resistance [6]. Instead, in this study, we propose to analyze all the SNPs near the target sites in 3′ UTR of all mRNA, to have a general overview of the SNPs' regulatory effect on the drug response.

We have developed a user-friendly online database, Mir2Drug (publicly accessible at http://bioinfo.njmu.edu.cn/Mir2Drug), which can help researchers to identify the SNPs, names as tarSNPs, which can affect drug response that does not only reside in target sites but also near target sites. We also identified those SNPs that indirectly affect drug response through protein-protein interaction (PPI) network and pathway. We show that the database's predictions can uncover most of the known experimentally supported cases and provide better performance than other existing databases and tools. Mir2Drug can be used to predict drug response to therapy and is useful for explaining the differences in drug response, discovery, and characterization of novel predictive and prognostic biomarkers.

## 2. Materials and Methods

### 2.1. Identifying SNPs Affected miRNA Regulation (tarSNPs)

Mature miRNA sequences were derived from the miRBase, release 21 [14]. SNPs that are located in the 3′ UTRs of all known genes and 3′ UTRs sequences were retrieved from UCSC Genome browser (dbSNP build 147 and NCBI build 38) [15]. For each SNP, we extracted the 30 bp flanking sequence of both upstream and downstream of the SNP in the 3′ UTR region of genes. Then, we assessed whether the two alleles of an SNP lead to different miRNA target sites based on the 61 bp DNA sequence, using the program PITA [16] with default parameters. PITA predicts potential miRNA targets using an estimated free energy. The SNPs changing the free energy between miRNA and DNA sequence were defined as tarSNPs.

The degree of binding is quantified using the PITA score change. Mathematically, the binding degree is described as

(1) $$\begin{array}{c} \text{diff}=T_{wt}-T_{var}, \end{array}$$

where *T*_wt_ represents the score of miRNA binding to the wild-type 61 bp DNA sequences using the program PITA and *T*_var_ represents the score of miRNA binding to the variant-type sequences. diff represents the degree of miRNA regulation change from wild-type allele to the variant-type; a positive diff represents a strengthen miRNA regulation ability from wild-type allele to the variant-type; on the contrary, a negative diff represents a weaken miRNA regulation ability. According to the binding of miRNA to the mRNA 3′ UTR, we assigned the potential tarSNPs to one of the four classes: “complete gain,” the mRNA acquires a new target site through the wild-type SNP into variant-type SNP; “complete loss,” mRNA loses a predicted target site through the wild-type SNP into variant-type SNP; “partial gain,” mRNA acquires more stable target site than without the SNP; “partial loss,” mRNA target site turns into instable target site with the SNP. For the scenario of multiple tarSNPs identified in a single patient, we utilize the normalized binding energy differences for prioritizing the tarSNPs, which is described as

(2) $$\begin{array}{c} \frac{\left|\text{diff}\right|}{\max\left(\left|T_{wt}\right|,\left|T_{var}\right|\right)}. \end{array}$$

### 2.2. Mapping Predicted tarSNPs for Direct Drug Target

We have already got all genes that contained at least one tarSNP. Then, we mapped the predicted genes to drug target and extracted the genes by drug target and by containing tarSNP, we got all targets that at least one tarSNP is physically located in it. These drug target genes that contained tarSNP defined direct drug target. All known associations of drug and targets were downloaded from the DrugBank database (DrugBank 5.0) [17]. We set all DrugBank targets containing tarSNP as the direct drug targets.

### 2.3. Network Integrating for Prediction of Indirect Drug Target

We extract all the genes that interact with direct drug target genes in PPI network and defined them as a PPIN-indirect drug target. Totally, 4234 PPIN-indirect drug target genes were identified. The human PPI data was derived from the HPRD database [18]. We also get all the genes that are located in the same pathway with direct drug target genes, we defined them as pathway-indirect drug target, we get 2124 pathway-indirect drug target genes in the metabolic pathway and 5424 in the nonmetabolic pathway, and we computed the length of shortest paths for each pathway-indirect drug target genes and drug target genes. The metabolic and nonmetabolic pathways were downloaded from the Reactome database [19]. To assist the identification of tarSNPs that can indirectly affect drug response, we mapped the predicted tarSNPs to indirect drug target and extracted the genes by indirect drug target and by containing tarSNP.

### 2.4. Mir2Drug Database Construction

All useful results and information in this work were organized into a set of relational MySQL tables for fast access. HTML, CSS, JavaScript, and PHP were used to construct the online website Mir2Drug which performs multiple browse and search functions running on Apache web server (http://www.apache.org).

## 3. Results

### 3.1. Functional miRNA Polymorphisms Widely Distributed on Drug Targets

**Figure 1:**
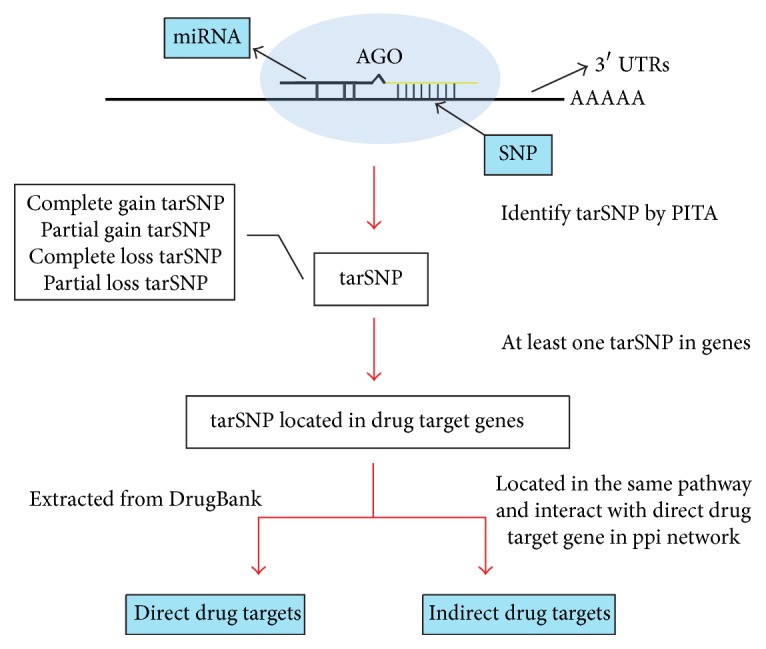
The workflow of the Mir2Drug database that identified SNPs affecting drug response.

Unlike other tools (PolymiRTS [10, 20], MicroSNiPer [12], MirSNP [21], and miRNASNP [22]), we considered that both SNPs reside and near the target site can affect the interaction of miRNA to the target site (tarSNPs). Therefore, we also include all genes that are located in the same pathway with direct drug target genes and that interact with direct drug target genes in PPI network as target genes (Figure 1). At least, we got direct 3408 drug target genes and 9143 indirect drug target genes; we got all targets that at least one tarSNP is located in it. These genes are candidate drug response genes underlying tarSNP regulation.

For a SNP in the miRNA target, if it gives rise to more free energy changed, more instability of the structure of the miRNA/mRNA interaction occurs. In addition, 5188 pairs of experimentally confirmed miRNA/mRNA interaction downloaded from miRTarBase [28]. And the average free energy of the structures on miRNA/mRNA interaction is −4.58 ± 3.60 kcal/mol. The free energy change of the structures is a relatively high level ranging from 1 to 33.71. About 50% of energy changes are >2.5 kcal/mol, which may affect the stability of the structure significantly. In our results, a set of 833134 out of 973671 SNPs were identified as tarSNPs on 10021 drug targets. The tarSNPs have been generally fallen into four classes based on our methods. TarSNPs of class “complete gain” and “partial gain” may cause a gain of miRNA function and downregulation of the target protein; if the miRNA target protein is a drug target, its decreased level will result in drug sensitivity and vice versa: tarSNPs of class “complete loss” and “partial loss” may cause a loss of miRNA regulation; it will cause upregulation of the target protein, resulting in drug resistance. Both direct drug targets and indirect drug targets are featured in the database.

### 3.2. SNPs in 3′ UTR of Drug Target Affect Drug Efficiency Induced by Gain or Loss Function of miRNA Targeting

Some drug target genes showed a significant downregulation or upregulation in the mRNA level in drug-treated cells comparing to the nontreated cells. We supposed that drug treatment may cause variation in the drug target genes' 3′ UTR to change the stability of the structure of miRNA/mRNA interaction. Some research showed that BTG1 was upregulated in response to treatment with tomato leaf extract (TLE) [23], recombinant bromelain [24], and insulin-like growth factor I (IGF-I) [25] in the MCF-7 breast cancer cell line, which contained a SNP (rs764927448) in BTG1 by query of the Cancer Cell Line Encyclopedia (CCLE). Moreover, we found that BTG1 loss is regulated by has-miR-1255b-5p through the wild-type SNP into variant-type SNP in Mir2Drug database (Table 1). Conversely, Tuna et al. showed that HER2/neu engages Akt to increase WT1 expression to stimulate S-phase proliferation and inhibit apoptosis in breast cancer cells, and then inhibition of HER2/neu with the anti-HER2/neu trastuzumab antibody decreased WT1 protein levels in HER2/neu-overexpressing BT-474 cells [26]. Ebada et al. showed that in vitro IC50 values of Norlichexanthone and anomalin A inhibited PIM1 in A2780 ovary cancer cell line [27]. We also found an SNP (rs746218880) in WT1 3′ UTR and an SNP (rs72552389) in PIM1 3′ UTR, which create miRNA/mRNA interaction in Mir2Drug database (Table 1).

### 3.3. Harnessing the Molecular Networks Mir2Drug Increased the Efficiency of Identifying Novel tarSNPs as well as Recapitulating Known tarSNPs

To demonstrate the effectiveness of our optimized method of recapitulating known cases where SNPs affected the miRNA binding, we manually compiled nine experimentally confirmed tarSNPs using text mining (Table 2). Although there are several existing databases predicting the gain or loss of targeting function of miRNA mediated by SNPs, such as PolymiRTS, miRNASNP, MicroSNiPer, and MirSNP, none of them evaluated the influences of tarSNPs on drug response alterations. In spite of the lack of large scale experimentally confirmed tarSNP data, notably, all nine only compared their performance with Mir2Drug using validated data from Table 2. All nine known tarSNPs confirmed in the literature had been predicted in our database.

From twelve cases, only four was corroborated in all four databases. Other four cases were corroborated no more than two databases; in our databases, they all have a certain degree of change in score. One case was not corroborated in all four databases, but it was predicted in our databases. The highest of the free energy change is 10.9 kcal/mol about rs2278414 in ZNF350 3′ UTR and has-miR-21-3p. And rs2278414: C > T was significantly associated with age-related cataract (ARC) risk, which associated with DNA double-strand break repair (DSBR) and nucleotide excision repair (NER) pathway [29].

### 3.4. Online Database Implementation

We have constructed a user-friendly web tool on genetic variations in miRNA target sites and their potential function in drug response. More specifically, an important focus of this study is to highlight the association of tarSNPs and drug response, thereby identifying tarSNPs that might possibly be involved in drug sensitivity and resistance. We presented a user-friendly website, Mir2Drug database, which will serve as the platform site to provide a practical resource of these drug target-related miRNA polymorphisms and their potential drug response alterations caused by target loss and gain information for all researchers and explore the association of variations in miRNA targets and cancer therapies efficacy and facilitate a mechanistic understanding of relationships among the genetic variations and drugs response. We packaged all the data into a MySQL database and built a user-friendly online website. The Mir2Drug database provides information from the two aspects: (1) drug response alterations medicated by miRNA polymorphisms in target 3′ UTR and (2) drug, pathway, and PPI information about drug targets. Mir2Drug supplies multiple functions for data browsing and searching by search gene symbol, SNP ID, miRNA ID, and an advanced search.

## 4. Discussion

In this study, we present a database, Mir2Drug, which provides comprehensive annotation information on genetic variations located in miRNA target sites belonging to drug target genes. We evaluate all SNPs in the 3′ UTRs, even if farther away from the miRNA target site, which can alter the miRNA regulation and hence would contribute to drug response. It is appreciated that most of the known cases can be rediscovered in our database. An important goal of this work is to identify the SNPs that can alter miRNA regulations and are also potentially associated with drug sensitivity and resistance in clinical trials. The database would be a valuable resource for experimentalists to explore the functional role of this class of SNP.

Although these SNPs are rare, they may be functionally important, because they can alter extensive mRNA expression by gain or loss of miRNA regulation. Recent studies have reported that genetic variations in miRNA processing genes and miRNA binding sites may affect the biogenesis of miRNA and the regulatory effect of miRNAs on their target genes and have a role in cancer development and treatment response [30–34]. However, a few of genes were found on the relationship between SNP in this gene and drug response. We have a new discovery that an SNP (rs751012151) in MRPL4 can be downregulated by hsa-miR-6089 and decrease drug resistance in our database (ΔΔ*G* = 45.54 kcal/mol), but so far it has no literature reported about the relationship between the MRPL4 and drug response. Future studies are necessary to explore the functional role of this class of SNPs. In addition, the known experimentally verified miRNA-disease associations were insufficient in this study. However, in many cases, such information would be very valuable. For example, we can collect this information to quantitatively compare the performance of existing tools (PolymiRTS, MicroSNiPer, MirSNP, and miRNASNP) using cross-validation method if we have sufficient experimentally verified cases and further optimize our method. With the accumulation of such validation and experimental confirmation of miRNA target interactions data, we plan to include this information in next version of our database; we would expect a much better annotation of Mir2Drug in the near future.

## Figures and Tables

**Table 1:** Drugs and their predicted differentially expressed targets in Mir2Drug database.

Drug	SNP ID	Gene symbol	miRNA	Wild free energy	Mutation free energy	ΔΔ*G* (kcal/mol)	Effect class	Literature
TLE	rs764927448	BTG1	hsa-miR-1255b-5p	−0.26	0.8	−1.06	Partial loss	[23]
Recombinant bromelain	rs764927448	BTG1	hsa-miR-1255b-5p	−0.26	0.8	−1.06	Partial loss	[24]
IGF-I	rs764927448	BTG1	hsa-miR-1255b-5p	−0.26	0.8	−1.06	Partial loss	[25]
Anti-HER2/neu trastuzumab antibody	rs746218880	WT1	hsa-miR-1193	None	−8.11	8.11	Complete gain	[26]
Norlichexanthone and anomalin A	rs72552389	PIM1	hsa-miR-7114-5p	None	−11.12	11.12	Complete gain	[27]

**Table 2:** Experimental validated miRNA polymorphisms and their predictions in Mir2Drug databases.

SNP id	Gene ID	Gene symbol	miRNA	Allele	Wild free Energy	Mutation free energy	ΔΔ*G* (kcal/mol)	Effect class	miRNASNP	PolymiRTS	MicroSNiPer	MirSNP
rs5186	185	AGTR1	hsa-miR-155-5p	A > C	−8.86	None	−8.86	Complete loss	Yes	Yes	Yes	Yes
rs4245739	4194	MDM4	hsa-miR-191-5p	C > A	−6.07	−0.098	−5.972	Partial loss	Yes	No	Yes	Yes
rs1434536	658	BMPR1B	hsa-miR-125b-5p	C > T	−7.27	−5.08	−2.19	Partial loss	No	No	Yes	Yes
rs3739008	4862	NPAS2	hsa-miR-17-5p	C > T	−9.56	−6.76	−2.8	Partial loss	Yes	Yes	No	Yes
rs3739008	4862	NPAS2	hsa-miR-519e-3p	C > T	−11.71	−8.91	−2.8	Partial loss	Yes	Yes	Yes	Yes
rs3739008	4862	NPAS2	hsa-miR-20b-5p	C > T	−7.49	−4.69	−2.8	Partial loss	Yes	Yes	No	Yes
rs1805672	10219	KLRG1	hsa-miR-584-5p	A > G	−6.9	None	−6.9	Complete loss	No	Yes	Yes	No
rs56109847	285242	HTR3E	hsa-miR-510-5p	G > A	−14.07	−9.69	−4.38	Partial loss	Yes	Yes	Yes	Yes
rs3203358	2626	GATA4	hsa-miR-583	C > G	−10.49	−5.98	−4.51	Partial loss	Yes	No	Yes	No
rs2278414	59348	ZNF350	hsa-miR-21-3p	C > T	None	−10.9	10.9	Complete gain	No	No	Yes	Yes
rs2278414	59348	ZNF350	hsa-miR-150-5p	C > T	−6.22	−10.96	4.74	Partial gain	Yes	Yes	Yes	Yes
rs868	7046	TGFBR1	hsa-let-7b-5p	A > G	−1.93	−0.24	−1.69	Partial loss	No	No	No	No
